# Disruption of the reciprocal inhibitory relationship between cell proliferation and melanogenesis in pet murine melanoma cells by ultraviolet radiation

**DOI:** 10.3389/fvets.2026.1826884

**Published:** 2026-07-27

**Authors:** Quanzhou Lv, Enchang Liang, Liangshuo Bai, Siqi Huang, Jiran Song, Hui Yin, Yan Li

**Affiliations:** 1 Pharmaceutical School, Jilin Medical University, Jilin, China; 2 Clinical Medicine School, Jiangxi University of Traditional Chinese Medicine, Nanchang, Jiangxi, China; 3 Clinical Laboratory School, Jilin Medical University, Jilin, China; 4 International Education School, Jilin Medical University, Jilin, China

**Keywords:** eumelanin, melanoma, PIAS3, proliferation, ultraviolet radiation

## Abstract

This study investigates the relationship between intracellular eumelanin content and proliferation of melanoma cells derived from pet murine under ultraviolet (UV) radiation. B16F10 cells, a murine melanoma cell line established from pet murine models, were exposed to UV doses ranging from 0 to 2,880 J/m^2^. Cell proliferation was assessed using the CCK-8 assay, while intracellular eumelanin content was quantified via microplate reader after cell lysis, normalized using BCA protein quantification. Linear regression was performed using partial regression analysis revealed a positive correlation between eumelanin content and cell proliferation at low-to-moderate UV doses. However, this linear relationship was disrupted at the highest dose. Protein–protein interaction (PPI) analysis further demonstrated that PIAS3, a regulatory protein, modulates both STAT3 and MITF, key factors in melanoma progression. These findings suggest that UV exposure enhances eumelanin production and proliferation in pet murine melanoma cells at sub-lethal doses, but excessive UV exposure may disrupt this balance, potentially through downregulation of PIAS3. The results highlight the dual role of UV radiation in promoting eumelanin synthesis as a protective mechanism while also driving tumor growth, offering insights into the pathogenesis of melanoma in pet murine and underscoring the importance of UV protection in their clinical management.

## 1 Background

Melanoma, a highly aggressive malignancy derived from melanocytes, remains one of the most challenging cancers to treat due to its rapid metastatic potential to regional lymph nodes and distant visceral organs (1). While human melanoma has been extensively studied, the unique biological characteristics of melanoma in small mammalian species, particularly in pet murine such as mice, rats, and hamsters, present a compelling yet underexplored research niche (2). These animals naturally develop melanocytic tumors that closely mirror human melanoma in terms of histopathological features and molecular complexity (3, 4).

Melanin is the core protective mechanism of skin against UV-induced damage, preventing cellular DNA damage through absorption and scattering of UV radiation as well as scavenging reactive ROS (5). Melanin is synthesized from tyrosine via tyrosinase-mediated catalysis and is classified into two types: eumelanin and pheomelanin (6). Eumelanin exhibits potent antioxidant activity, whereas pheomelanin synthesis is accompanied by ROS production and demonstrates pro-oxidant properties (7). As an enzyme-catalyzed oxidation reaction, melanin biosynthesis can generate ROS, including superoxide anions and hydrogen peroxide, thereby triggering lipid peroxidation, inhibition of protein function, and oxidative DNA damage (8, 9). ROS can induce formation of 8-hydroxyguanine, leading to genomic instability and cytotoxicity (10, 11). In patients with vitiligo, due to melanocyte deficiency, somatic mutation burden is reduced, resulting in significantly decreased risks of melanoma and non-melanoma skin cancers (12). A critical distinction between human and rodent melanomas lies in their relationship with eumelanin synthesis (13). UV radiation not only acts as a primary carcinogen but also stimulates eumelanin production through MITF-mediated tyrosinase activation (14). The classical hypothesis posits that high eumelanin content suppresses malignant transformation by scavenging reactive oxygen species and inhibiting cell proliferation (15, 16). However, our preliminary studies in pet murine such as Cricetidae and Muridae species have revealed a paradoxical phenomenon: melanoma cells with elevated eumelanin levels exhibit enhanced metastatic potential and resistance to apoptosis, challenging the long-held inverse relationship between eumelanin content and tumor aggressiveness (17, 18).

The JAK/STAT3 signaling cascade plays a central role in this complex interplay. Constitutive activation of STAT3, a well-established oncogene in melanoma, drives cell proliferation and survival through transcriptional activation of genes like Bcl-2 and cyclin D1 (19). However, the melanogenic transcription factor MITF, which directly regulates tyrosinase expression and eumelanin biosynthesis, appears to exist in a dynamic equilibrium with STAT3 (20). Clinical data from human patients demonstrate a negative correlation between STAT3 and MITF expression (21), yet our rodent studies reveal a more nuanced relationship where both pathways can co-exist in a context-dependent manner. This discrepancy highlights the need for species-specific investigations into the molecular crosstalk between these pathways.

Protein Inhibitor of Activated STAT3 (PIAS3) emerges as a key regulatory node in this network. While PIAS3 is classically known to inhibit STAT3 activity through SUMOylation and DNA binding suppression (22), our findings in pet murine suggest an expanded role for PIAS3 in melanoma biology. Notably, we observe that PIAS3 not only suppresses STAT3-driven proliferation but also directly interacts with MITF to modulate eumelanin synthesis. This dual inhibitory function is further complicated by evidence of PIAS3 autoinhibition, where the protein’s own structural dynamics may limit its effectiveness in tumor suppression. Additionally, emerging data indicate that cross-talk between the STAT3-MITF axis and other signaling pathways, such as WNT5A-mediated non-canonical Wnt signaling and C/EBP transcription factors may create a complex regulatory lattice that governs melanoma progression in these animals (23).

The significance of this research is underscored by the growing clinical relevance of pet murine melanomas. By elucidating the molecular mechanisms underlying the STAT3-MITF crosstalk in pet murine, our work may advance the treatment of this specific animal cancer. In normal melanocytes, a small fraction of ROS released during melanin synthesis is rapidly cleared by the cellular antioxidant network, with antioxidant activity exerting absolute dominance. In the melanoma microenvironment, cancer cells enhance ROS generation through RAS signaling pathways and glycolysis while upregulating antioxidant systems to maintain ROS at sublethal levels (24), promoting tumor survival and metastasis. Furthermore, melanin’s high antioxidant capacity confers resistance of melanoma cells to radiotherapy and chemotherapy, with melanin levels negatively correlated with radiotherapy efficacy (25). A phenotype characterized by high proliferative activity and elevated melanin levels may represent a compensatory protective mechanism against oxidative stress. Our preliminary data suggesting that eumelanin synthesis and tumor aggressiveness may be positively correlated in these models challenge fundamental assumptions about melanoma pathogenesis, opening new avenues for therapeutic intervention targeting both pigmentation and oncogenic signaling pathways.

## 2 Materials and methods

### 2.1 Chemicals and materials

The B16F10 cell line was obtained from Procell. The CCK-8 assay kit was purchased from Xinsemai Biotechnology Co., Ltd. The BCA protein quantification kit was sourced from Beyotime Biotechnology. DMEM high-glucose medium was obtained from HyClone (United States). Fetal bovine serum (FBS) was supplied by Zhiqian Biological Products Co., Ltd. Dimethyl sulfoxide (DMSO) was purchased from Beyotime Biotechnology. The cell culture incubator was produced by Beijing Oriental Anruo Biochemical Technology Co., Ltd. The laminar flow hood was manufactured by Suzhou Purification Instrument Factory. The inverted microscope was supplied by Olympus (Japan). Cell culture dishes were produced by JET BioScience (Canada). The 24-well plates were obtained from Corning (United States). The dual-fluorescence confocal microscope was a product of Olympus (Japan).

### 2.2 Cell culture

B16F10 cells were cultured in DMEM medium supplemented with 10% FBS, 100 U/mL penicillin, and 100 μg/mL streptomycin. Cells were maintained in a humidified incubator at 37 °C with 5% CO_2_. The medium was replaced every 2 days. When cells reached 80–90% confluence, they were digested with 0.25% trypsin and passaged.

### 2.3 UV irradiation protocol

Cells were rinsed twice with PBS, followed by the addition of a thin layer of PBS to prevent dehydration. UV irradiation was performed using a 36 W UV lamp under a laminar flow hood. The irradiation dose was calculated as 24 J/m^2^·s based on the irradiation area. Cells were exposed for 15 s, 30 s, 45 s, 60 s, or 120 s. After irradiation, serum-free medium was added, and cells were cultured for an additional 24 h.

### 2.4 Cell proliferation assay (CCK-8)

Log-phase B16F10 cells were digested with 0.25% trypsin, resuspended at 5 × 105 cells/mL, and seeded in 6-well plates at 2 mL per well. Cells were cultured for 24 h under standard conditions. Following UV irradiation with three replicates per group, cells were incubated for an additional 24 h. CCK-8 reagent was added according to the manufacturer’s instructions, and cells were further incubated for 2 h. Absorbance was measured at 450 nm.

### 2.5 Calcein-AM assay for cell proliferative viability

Cells were seeded in culture plates and cultured under routine conditions until adherent, followed by exposure to different doses of ultraviolet irradiation. After irradiation, the culture medium was discarded, and serum-free medium containing 5 μmol/L Calcein-AM was added. The plates were incubated at 37 °C with 5% CO_2_ for 30 min in the dark. Fluorescence intensity was measured using a fluorescence microplate reader with an excitation wavelength of 485 nm and an emission wavelength of 520 nm. Fluorescent signals were continuously recorded within a detection time window of 0–60 s, and the fluorescence values were used to represent cell proliferative viability. Five replicate wells were set for each group, and the experiment was independently repeated three times.

### 2.6 Intracellular eumelanin content assay

Log-phase B16F10 cells were processed as in the CCK-8 assay. After UV irradiation and 24 h of post-treatment culture, cells were collected, lysed, and centrifuged at 12,000 rpm for 10 min. The eumelanin-containing pellet was resuspended in 1 mol/L NaOH containing 10% DMSO and incubated at 80 °C for 45 min. Eumelanin content was quantified by measuring absorbance at 405 nm.

### 2.7 BCA protein quantification

The supernatant from the eumelanin assay was used for protein quantification. Samples were processed according to the BCA kit instructions. A 20 μL aliquot of each sample was incubated at 37 °C for 40 min, and protein concentration was determined by measuring absorbance at 562 nm. A standard curve was generated to calculate sample protein content.

### 2.8 PPI analysis

STRING database^1^ (26) was employed to investigate interactions between STAT3 and MITF. The network was expanded to identify shared regulators and explore potential mechanisms of signal crosstalk between STAT3 and MITF.

### 2.9 Western blot analysis of PIAS3 expression

Cells were treated with different doses of UV irradiation for 15 to 60 s. After treatment, total protein was extracted using RIPA lysis buffer containing protease and phosphatase inhibitors. Protein concentration was determined using a BCA protein assay kit. Equal amounts of protein samples were separated by 10% SDS-PAGE and transferred onto PVDF membranes. The membranes were blocked with 5% non-fat milk at room temperature for 1 h and then incubated with primary antibody against PIAS3 overnight at 4 °C. After washing, the membranes were incubated with HRP-conjugated secondary antibody at room temperature for 1 h. Protein bands were visualized using an ECL detection system. *β*-actin was used as an internal control. The relative expression level of PIAS3 was analyzed by ImageJ software. Results showed that PIAS3 expression was gradually decreased with increasing UV dose within 15–60 s.

### 2.10 Co-IP assay for PIAS3-MITF interaction

To explore the regulatory relationship between PIAS3 and MITF, Co-IP was performed *in vitro*. Cells were lysed in IP lysis buffer, and the supernatant was collected after centrifugation. Input samples were reserved, and the remaining lysate was incubated with anti-PIAS3 antibody or anti-MITF antibody overnight at 4 °C with gentle rotation. Protein A/G agarose beads were added and incubated for 4 h at 4 °C. The beads were washed repeatedly with IP buffer, and the bound proteins were eluted by loading buffer. The interaction between PIAS3 and MITF was detected by Western Blot. The results confirmed that PIAS3 could physically interact with MITF *in vitro*.

### 2.11 Statistical analysis

Data analysis was performed using R packages. Comparisons between groups were conducted using Student’s *t*-test. Linear regression was performed using partial regression fitting methods.

## 3 Results

### 3.1 UV irradiation promotes cell proliferation in a dose-dependent manner

**Figure 1:**
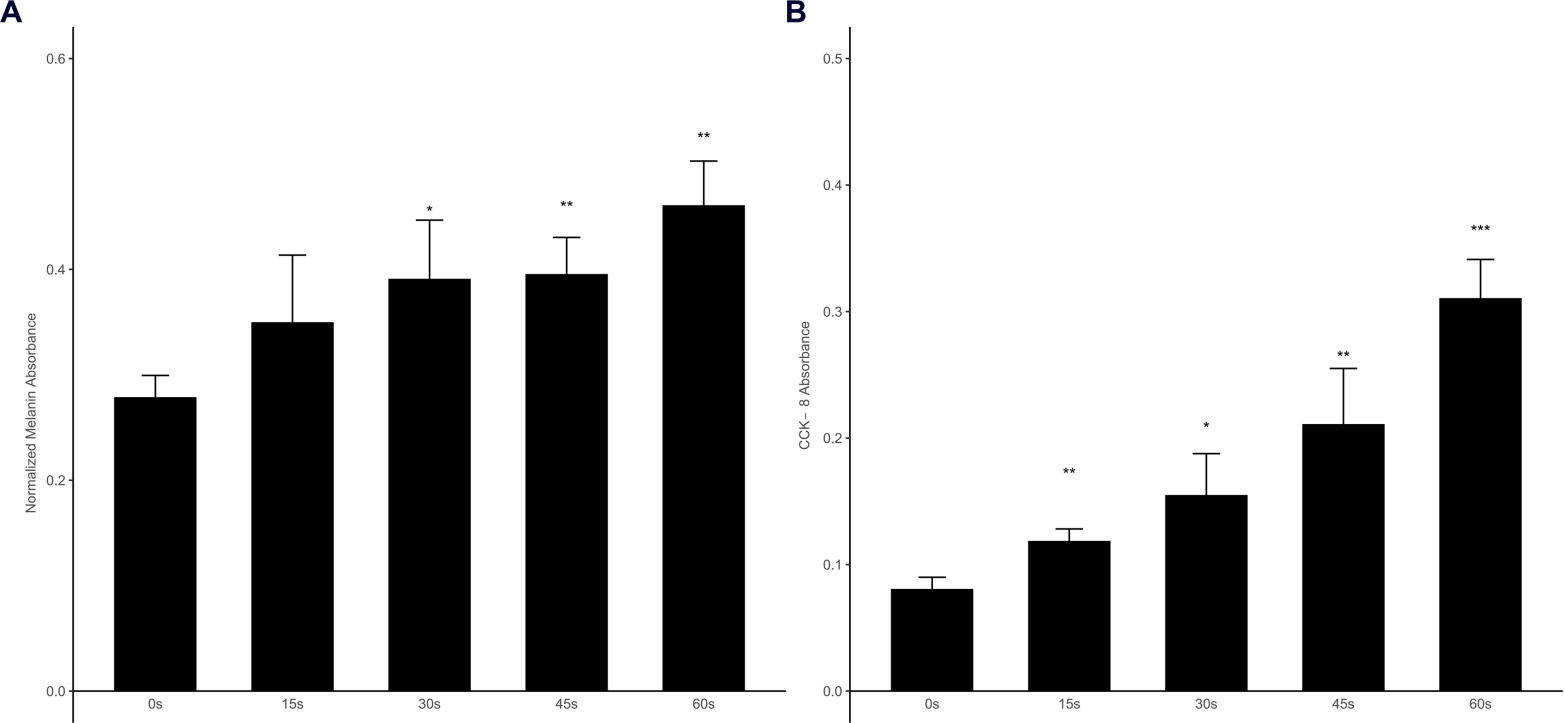
**(A)** Assessment of cell viability via CCK-8 assay after UV exposure. The y-axis shows the CCK-8 absorbance with pre-subtracted plate background absorbance, and the *x*-axis displays the gradient duration of UV irradiation. Data are presented as mean ± standard deviation. **(B)** Assessment of eumelanin production in cells after UV exposure. The y-axis shows the eumelanin absorbance with pre-subtracted plate background absorbance and normalized to total cellular protein content, and the x-axis displays the gradient duration of UV irradiation. Data are presented as mean ± standard deviation.

We first examined changes in B16F10 cell viability following exposure to different UV doses. Results showed that, compared to the blank control, cell viability increased progressively with UV exposure duration (0–60 s, equivalent to 0–1,440 J/m^2^) (Figure 1A). To mitigate potential interference of mitochondrial dysfunction on cell proliferation assessment, we independently evaluated cell proliferation activity using the Calcein-AM assay, which demonstrates low cytotoxicity. Results showed that cell proliferation activity increased with increasing UV concentration during 0–60 s of irradiation.

### 3.2 UV irradiation enhances intracellular eumelanin synthesis in a dose-dependent manner

Typically, increased melanoma cell viability correlates with suppressed eumelanin synthesis. To investigate this relationship under UV exposure, we normalized eumelanin content by measuring protein levels per well, accounting for varying proliferation rates. Interestingly, compared to the blank control, eumelanin content increased with UV exposure duration (0–60 s) (Figure 1B).

### 3.3 Cell proliferation rate positively correlates with intracellular eumelanin content under UV exposure

**Figure 2:**
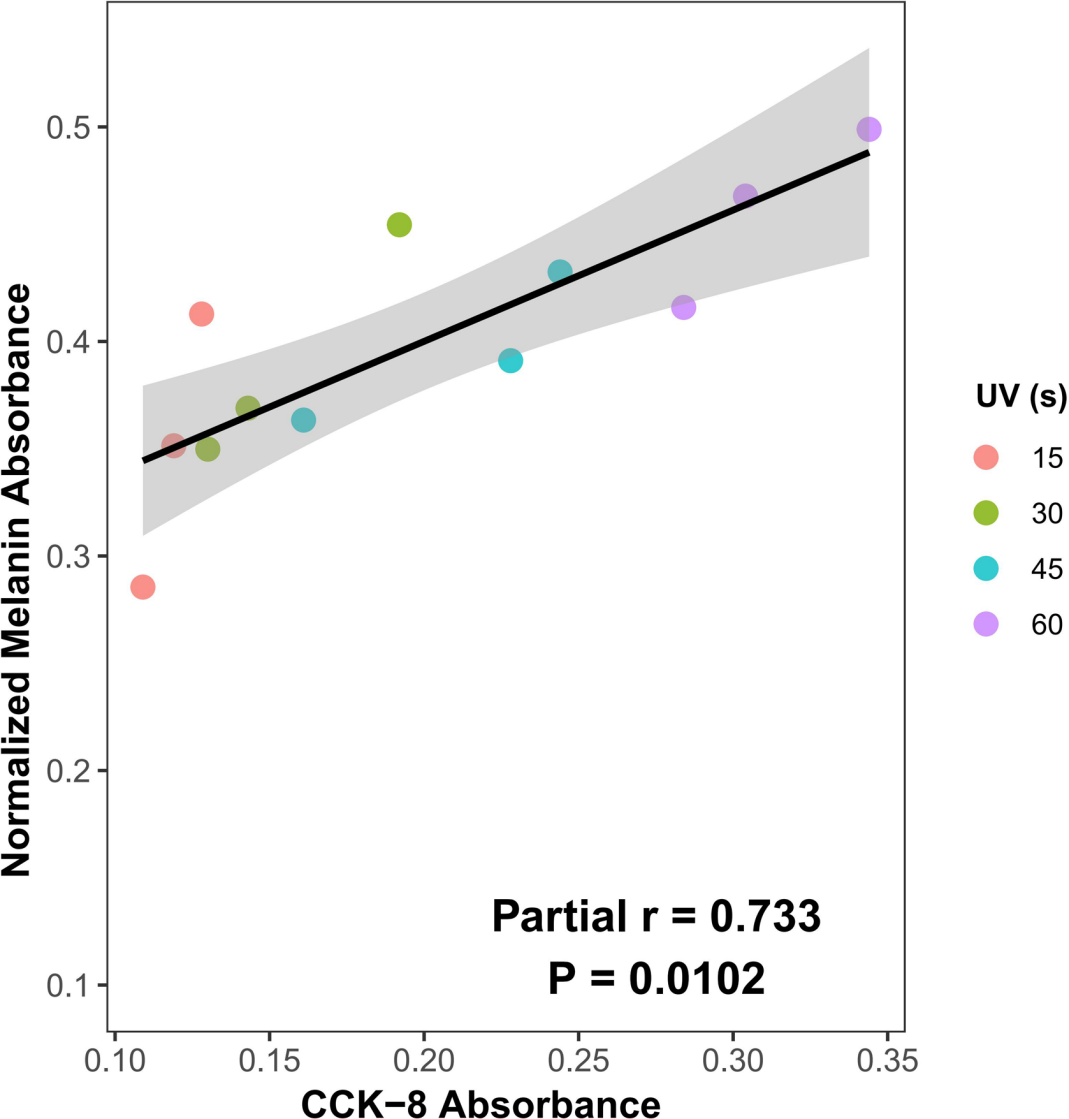
The plot depicts the relationship between eumelanin concentration and cell proliferation after statistically controlling for covariates. Each point represents an independent biological replicate. The solid regression line indicates a significant positive partial effect (*p* < 0.05).

We observed a potential relationship between cell viability and eumelanin content after UV irradiation. Traditional linear regression may distort results due to varying UV doses; thus, partial regression analysis was employed. Results revealed a positive correlation between proliferation rate and eumelanin content (*R* = 0.733, *p* = 0.0102), though the correlation strength was moderate (Figure 2).

### 3.4 PIAS3 may act as a shared regulator of STAT3 and MITF

**Figure 3:**
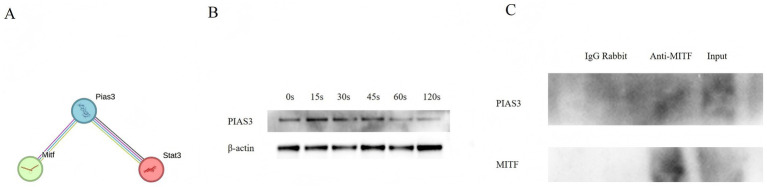
**(A)** The PPI network was constructed based on the STRING database. Nodes represent the target proteins PIAS3, STAT3, and MITF, respectively. Color-coded lines indicate different types of interaction evidence: blue lines for annotated interactions curated from professional databases, purple lines for interactions validated by experimental data, green lines for interactions co-mentioned in the same text of published literature, and black lines for co-expression evidence between two proteins. **(B)** B16F10 cells were exposed to UV irradiation for the indicated time periods (0, 15, 30, 45, 60 and 120 s). Cell lysates were subjected to Western blot analysis using antibodies against PIAS3 and *β*-actin. *β*-actin served as the loading control. The protein level of PIAS3 gradually decreased over time, while *β*-actin remained unchanged. **(C)** Co-IP assay verified the *in vitro* binding between PIAS3 and MITF. Total cellular protein lysates were immunoprecipitated with anti-MITF antibody and control IgG antibody. Input represented whole-cell lysates without immunoprecipitation. The co-precipitated proteins were detected via western blot, which confirmed the physical interaction between PIAS3 and MITF in vitro.

To elucidate the molecular mechanism linking cell viability and eumelanin content, we performed PPI analysis. STAT3, a key regulator of melanoma proliferation, and MITF, which modulates eumelanin synthesis via tyrosinase activity, were analyzed using the STRING database. Results indicated that a potential interaction may exist between STAT3 and MITF, with PIAS3 identified as a shared regulatory node in the expanded interaction network (Figure 3). Based on this, Western blot analysis was used to examine PIAS3 protein expression under varying UV irradiation conditions. Results showed that PIAS3 expression levels decreased progressively within the 15–60 s irradiation range as UV intensity increased. To further investigate the interaction between PIAS3 and MITF, Co-IP was performed, revealing that PIAS3 binds to MITF *in vitro*.

### 3.5 The positive correlation between cell viability and eumelanin content is disrupted at high UV doses

**Figure 4:**
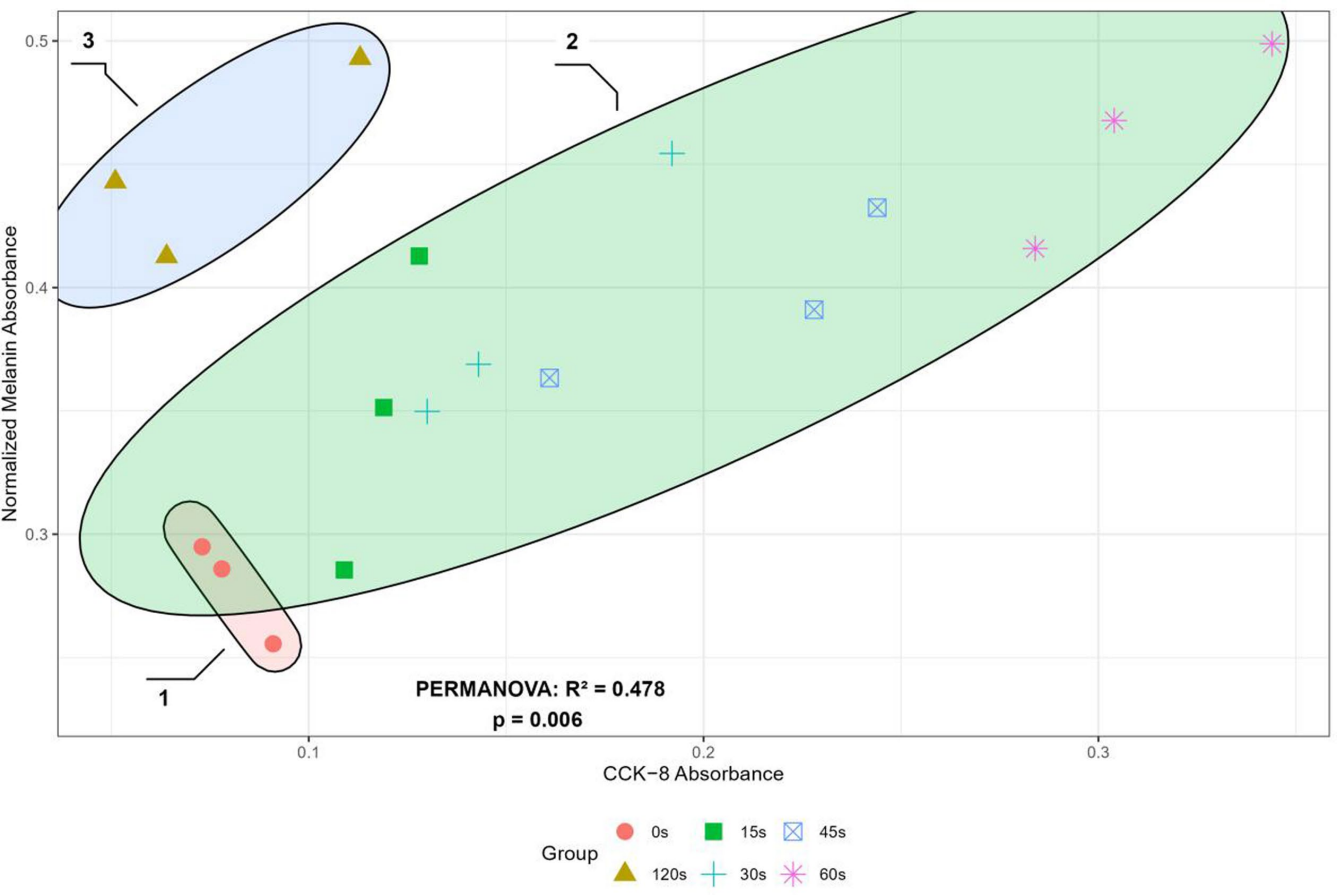
Unsupervised clustering analysis of samples under gradient UV irradiation. Samples in the 0 s UV irradiation group and 120 s UV irradiation group formed two independent clusters respectively, while samples from the remaining UV irradiation time points were clustered into one single cluster. The differences in clustering distribution among groups were statistically significant (*p* < 0.01).

We further tested whether the positive correlation persisted under high UV exposure (Wavelength range: 320–400 nm). At 120 s (equivalent to 2,880 J/m^2^), cell viability decreased while eumelanin content increased, disrupting the previously observed positive relationship compared to the control group (Figure 4). Western Blot analysis showed that PIAS3 expression was upregulated after 120 s of UV irradiation.

## 4 Discussion

Laboratory-bred hamsters are prone to melanoma (27), and the underlying mechanisms of melanoma development remain incompletely understood. Long-term UV exposure is recognized as a risk factor for melanoma progression (28). UV irradiation can trigger TP53 or PTCH mutations to accelerate BRAF-driven melanoma development (29). Concurrently, DNA damage, oxidative stress, and inflammation induced by UV exposure may counterintuitively suppress tumor growth (30). However, a moderate environmental stress, such as mild oxidative stress from external factors, enhances cell survival and proliferation by activating the JAK/STAT and SRC/EGFR pathways via ROS elevation (31). Additionally, melanoma, characterized by microsatellite instability, frequently undergoes rapid genetic mutations during proliferation, enabling rapid selection of subclones with growth advantages (32).

Our results demonstrate that UV exposure doses of 0–1,440 J/m^2^ significantly increase tumor cell viability in a dose-dependent manner, suggesting a promotion of survival and proliferation under low-dose UV. Notably, at 1,440 J/m^2^, although cellular proliferation continues to rise, morphological abnormalities emerge, potentially reflecting UV-induced subcellular structural damage. To explore higher doses, we tested cellular proliferation at 2,880 J/m^2^ and observed a suppressed activity, indicating that excessive UV exposure reverses its pro-survival effect.

UV radiation directly stimulates eumelanin synthesis by activating tyrosinase (33) or upstream signaling pathways like MAPK (34) and FOXO1 (35) in melanocytes. Additionally, UV-induced damage to keratinocytes promotes *α*-melanocyte-stimulating hormone (α-MSH) secretion, which activates eumelanin production via the cAMP/PKA/CREB/MITF cascade (36). Melanoma cells maintaining pigmentation function exhibit enhanced eumelanin synthesis under environmental stimuli (37). In our investigations using the B16F10 cell line, UV exposure up to 1,440 J/m^2^ caused a dose-dependent increase in intracellular eumelanin levels. Even at 2,880 J/m^2^, where cell proliferation is inhibited, eumelanin content remained elevated compared to non-irradiated controls Table 1.

**Table 1:** Cell proliferation (Calcein-AM, ×100000).

Group	Mean	SD	*P*
0 s	0.440	0.026	
15 s	0.512	0.031	<0.001
30 s	0.736	0.032	<0.0001
45 s	1.056	0.081	<0.0001
60 s	1.254	0.044	<0.0001

Clinical and animal model studies reveal an inverse relationship between melanoma cell proliferation and eumelanin synthesis (18, 38). However, our analysis excluding UV effects revealed a weak yet statistically significant positive correlation between eumelanin levels and proliferation rates. This suggests a complex regulatory mechanism governing these parameters. UV radiation may bridge this interaction through dual effects: promoting eumelanin secretion in a dose-dependent manner while binding eumelanin synthesis and proliferation in a UV-dose-independent manner under moderate exposure. This dual mechanism explains the low correlation coefficient in statistical analyses, though the relationship persists with significance. The dissociation of eumelanin and proliferation at high UV doses further supports this hypothesis.

STAT3 is a central transcription factor of the JAK–STAT signaling pathway, which is constitutively activated across multiple malignancies (39). In primary and metastatic melanoma tissues, STAT3 activation levels are significantly elevated, whereas in normal melanocytes or benign lesions, STAT3 remains largely inactive, indicating its role as a key oncogene driving melanoma progression (40). Mechanistically, upstream kinases such as Src phosphorylate STAT3 at Tyr705, promoting homodimerization and nuclear translocation to exert transcriptional activity (41). STAT1 primarily mediates anti-tumor immune responses (42), whereas STAT3 promotes cell survival, proliferation, metastasis, and immune evasion (43), with both showing functional antagonism and rare co-expression (40). Compared to other STAT family members, such as STAT5, that function in limited tumor stages, STAT3 broadly participates in multiple melanoma development phases, making it the focus of this study. MITF is the master transcription factor regulating melanocyte development, differentiation, survival, and pigmentation (44). PIAS3 simultaneously inhibits both STAT3 and MITF by binding to STAT3 to block its transcriptional activation capacity while interacting with MITF to suppress its activity, forming an MITF-PIAS3-STAT3 regulatory network (45, 46). PPI network analysis confirms STAT3 and MITF as core hub factors connecting melanin synthesis with cellular proliferation. Previous studies corroborate PIAS3’s dual inhibitory role (47), and our findings suggest that UV downregulates PIAS3. This likely releases STAT3’s proliferative potential and MITF’s eumelaninogenic activity, thereby coupling proliferation and eumelanin synthesis until high-dose UV disrupts this balance. So this study focuses on the signaling crosstalk between STAT3 and MITF within the JAK–STAT pathway, investigating its potential role in regulating melanin synthesis. Subsequent research will extend to PIAS3 and other MITF regulators, including STAT family members such as STAT1, to elucidate the comprehensive effects of this signaling axis on pigmentation biology and tumor behavior.

## 5 Conclusion

In UV-irradiated melanoma cells, moderate UV exposure enhances both cell viability and eumelanin content in a dose-dependent manner, with a statistically significant correlation between these parameters. This association likely involves UV-mediated PIAS3 suppression, unleashing proliferative and melanogenic pathways. However, excessive UV exposure disrupts this synergistic relationship, demonstrating a dual threshold-dependent effect on melanoma biology. These findings highlight the complex interplay between environmental stimuli and tumor biology, with PIAS3 as a potential regulatory nexus.

## Data Availability

The raw data supporting the conclusions of this article will be made available by the authors, without undue reservation.

## Footnotes

1
https://cn.string-db.org
